# Decoding Fibroblast Heterogeneity in Osteoarthritis: Identification of a Fibrosis-Associated Subtype and Novel Diagnostic Biomarkers

**DOI:** 10.1155/mi/7066432

**Published:** 2025-09-23

**Authors:** Qingpeng Sun, Feng Niu, Li Wang, Honglin Pi, Chongtao Han, Jun Gao

**Affiliations:** ^1^Department of Orthopedics, Xiangyang Hospital of Traditional Chinese Medicine, Hubei University of Chinese Medicine, 24 Changzheng Road, Xiangyang, Hubei Province 441000, China; ^2^Department of Orthopedics, The Third Affiliated Hospital of Henan University of Chinese Medicine, 63 Dongming Road, Zhengzhou, Henan Province 450003, China

**Keywords:** cell clusters, diagnostic, fibroblasts, osteoarthritis, single-cell RNA-sequencing

## Abstract

**Background:** Fibroblasts are key contributors to extracellular matrix remodeling and fibrosis, thereby playing a crucial role in the pathogenesis of osteoarthritis (OA). However, their heterogeneity and functional subtypes in OA remain poorly understood.

**Methods:** The single-cell RNA sequencing (scRNA-seq) data of OA and two independent datasets were downloaded from the Gene Expression Omnibus (GEO) database. Subsequently, the Seurat package was utilized to normalize and downscale the scRNA-seq data and classify different cell types. Gene set enrichment analysis (GSEA) was applied to identify significantly enriched biological processes (BPs) specific in each cell cluster. Employing the Monocle2 package, the progression trajectories of OA were analyzed based on the dynamic gene expression changes in the fibroblast subtypes. Finally, single sample GSEA (ssGSEA) and differential expression analysis were combined to screen diagnostic biomarkers for OA, and their diagnostic efficacy was assessed by receiver operating characteristic (ROC) curves and principal component analysis (PCA).

**Results:** Using the scRNA-seq data of OA samples, we identified five different cell types (fibroblasts, endothelial cells [ECs], lymphoid cells, mural cells, and myeloid cells), with fibroblasts accounting for the highest proportion. Then, we found that Fibroblast subtype 3 was notably enriched in fibrosis-related pathways. The pseudotime trajectory analysis showed that genes associated with extracellular matrix and cell adhesion were significantly upregulated during the transformation from healthy status to OA. Additionally, the enrichment score of Fibroblast 3 in the OA tissue was higher than that in healthy tissue, which indicated that fibroblast 3 may promote the development of OA. Finally, four genes (*MTUS2*, *GPR1*, *GABRA4*, and *SGCA*) with strong diagnostic performance were identified as the biomarkers for OA.

**Conclusion:** The fibroblast subtypes identified by the present research played a critical role in the pathogenesis of OA, and the four biomarkers may serve as new targets for the early diagnosis and treatment of OA.

## 1. Introduction

Osteoarthritis (OA) is a joint disease characterized by gradual degradation of joint cartilage and changes in the surrounding bone tissues. OA affects patients across different age groups, particularly the elderly and those with joint injuries or overuse of joint [1–3]. Clinically, OA presents as joint pain, stiffness, and functional impairment, often leading to significant disability at advanced stages [4]. According to the Global Burden of Disease Study, the incidence of OA has increased from approximately 247 million cases in 1990 to over 527 million in 2019, with a particularly high prevalence in adults aged ≥60 years [5–7]. At present, early-stage OA remains difficult to be diagnosed accurately. The Kellgren–Lawrence (KL) grading system based on joint space narrowing and sclerosis is the most common radiographic method, with magnetic resonance imaging (MRI) providing additional structural details [8]. However, a frequent mismatch between imaging findings and symptoms often complicates clinical evaluation. Specifically, some patients exhibit severe radiographic changes yet remain asymptomatic, whereas others report pain despite minimal imaging evidence [9]. Currently, we face a lack of specific and reliable biomarkers for the early diagnosis of OA.

In OA, fibroblasts are the major mesenchymal cell type in synovial tissues and are involved in extracellular matrix synthesis, remodeling, and inflammatory response regulation [10, 11]. Studies have shown that OA-associated fibroblasts can promote synovial fibrosis and cartilage degradation through the secretion of collagen, matrix metalloproteinases, and inflammatory factors, thereby driving the disease progression [12]. In addition to forming the structural framework of the synovium, tissue-resident fibroblast-like synoviocytes play a pivotal role in regulating macrophage metabolism [13]. However, current knowledge of the heterogeneity of fibroblasts in OA is still limited, in particular, we lack a systematic analysis of their dynamic evolution. Therefore, classifying key fibroblast subtypes and their molecular characteristics not only helps clarify their role in OA progression but can also provide novel potential targets for the diagnosis of OA and its targeted therapy. The single-cell RNA sequencing (scRNA-seq) is a high-resolution technique for studying the gene expression of individual cells and revealing the interactions between different cell types and states [7, 14]. Previous studies utilizing scRNA-seq data have revealed the spatial and transcriptional landscapes of OA synovium and identified key factors that drive spatial homeostasis [15]. Here, this study classified fibroblast subtypes and discovered diagnostic biomarkers involved in the development of OA based on the scRNA-seq data from OA patients and healthy samples and two independent datasets. Computational methods were used for cell clustering and identification of marker genes for each cell cluster. Gene set enrichment analysis (GSEA) was applied to analyze significantly enriched biological processes (BPs) specific to each cell cluster. The pseudotime trajectory analysis was performed to track the expression of genes across key cell clusters during OA development. Enrichment scores were calculated to identify diagnostic biomarkers for OA. These findings provided potential therapeutic targets for OA treatment, contributing to the understanding of OA pathogenesis and prevention of disease progression.

## 2. Materials and Methods

### 2.1. Data Acquisition and Preprocessing

The scRNA-seq data of GSE216651 containing three OA samples and three healthy samples, were acquired from the Gene Expression Omnibus (GEO) database (https://www.ncbi.nlm.nih.gov/geo/query/acc.cgi?acc=GSE216651). The scRNA-seq data were filtered under the criteria that each gene was expressed in at least three cells and each cell expressed at least 200 genes. Then, the proportion of mitochondrion and rRNA was calculated using the PercentageFeatureSet function in the Seurat R package [16], ensuring that each cell expressed a gene number between 200 and 5000 and the content of the mitochondrion gene was below 20%. The data were normalized using the NormalizeData function. Next, the ScaleData function was applied to scale all the genes from the six samples, followed by performing principal component analysis (PCA) for dimensionality reduction. After confirming that the data variability was captured by the first 20 PCs, the anchor point at dim = 20 was determined, and batch effects between samples were eliminated using the Harmony R package (parameters: lambda = 0.5 and max.iter.harmony = 30) [17]. Cell clustering analysis was performed at the resolution of 0.1, while clustering analysis of specific fibroblasts was conducted at the resolution of 0.3.

To further expand the sample size for the validation analysis, GSE55457 and GSE55235 datasets containing 20 OA samples and 20 control samples were merged to validate the reliability of the identified diagnostic markers. Subsequently, we converted probe IDs to gene symbols based on the GPL96 platform annotation information and used ComBat of the sva package to remove batch effects with the following parameter settings: par.prior = TRUE, prior.plots = FALSE, mean.only = FALSE, ref.batch = NULL, BPPARAM = bpparam(“SerialParam”) [18].

### 2.2. Clustering Analysis and Recognition of Signature Genes in the Cell Clusters

Uniform manifold approximation and projection (UMAP) analysis was further employed for dimensionality reduction of the scRNA-seq data of GSE216651, and the results of clustering were visualized by the RunUMAP function [19]. Cell clustering was performed using the FindNeighbors function and FindClusters function at the resolution of 0.1 for all OA cells and at 0.3 for fibroblasts. Subsequently, the marker genes from the CellMarker2.0 database (http://bio-bigdata.hrbmu.edu.cn/CellMarker/) were utilized to annotate the cell clusters. The FindAllMarkers function was applied to recognize high-expressed genes in each cell cluster under the criteria of logfc.threshold = 0.25, min.pct = 0.25, and only.pos = T.

### 2.3. GSEA

GSEA was performed based on the scRNA-seq data of GSE216651. First, the differentially expressed genes (DEGs) between the OA and healthy samples were identified using the FindMarkers function (|logFC| > 0.1). Then, Gene Ontology (GO) enrichment analysis was conducted using the gseGO function in the clusterProfiler R package [20] to identify differentially enriched BPs between OA and healthy samples and those enriched in fibroblasts (parameters: pvalueCutoff = 0.05, pAdjustMethod = “BH”).

### 2.4. Pseudo-Time Trajectory Analysis

To reveal the role of specific fibroblast subtypes and dynamic gene expression changes during OA progression, the Monocle2 R package was used to perform pseudotime trajectory analysis on the key cell clusters [21]. Healthy and OA tissues were considered as two distinct cellular states for constructing cellular developmental trajectories. Specifically, healthy tissues were regarded as the beginning state, while the cells in OA tissues were regarded as the endpoint. First, the newCellDataset function was applied to establish the Monocle object, the reduceDimensional function was employed for dimensionality reduction (parameters: max_components = 2 and method = "DDRTree"). The orderCells function was utilized to sort the cells and construct trajectories. Next, the expression changes of genes associated with the pseudotime during OA development were analyzed applying differentialGeneTset function. The plot_pseudotime_heatmap function and plot_genes_in_pseudotime function were employed to visualize the heatmap and scatterplot of pseudotime-relevant genes, respectively.

### 2.5. Identification of Diagnostic Biomarkers for OA

The combined dataset (GSE55457 and GSE55235 datasets) was employed to validate the role of key cell clusters in OA development. The enrichment scores of key cell clusters in independent datasets were calculated based on the marker genes by single sample GSEA (ssGSEA) using the GSVA R package [22]. The DEGs (|log_2_FoldChange(FC)|>log_2_(1) and *p*.adjusted < 0.05) between the OA and healthy samples in independent datasets were identified using the limma R package [23] and visualized into a volcano plot. Then, the upregulated DEGs were intersected with the marker genes in the key cell clusters to obtain diagnostic biomarkers of OA. Further, the diagnostic performance of the biomarkers was evaluated in independent datasets according to the receiver operating characteristic (ROC) curve using the timeROC R package [24]. PCA was performed to assess the ability of the cell clusters in distinguishing between OA tissue and normal tissue.

### 2.6. Statistical Analysis

All statistical data and plots were analyzed and generated in the R language (version 4.3.1). A Z-test for proportions was used to compare significant differences in the cellular proportions of individual cells in the two groups. The Wilcoxon rank test was applied to compare the differences between two groups. A *p* < 0.05 denoted a statistically significant difference.

## 3. Results

### 3.1. The Highest Proportion of Fibroblasts Was Detected in OA Patients

**Figure 1:**
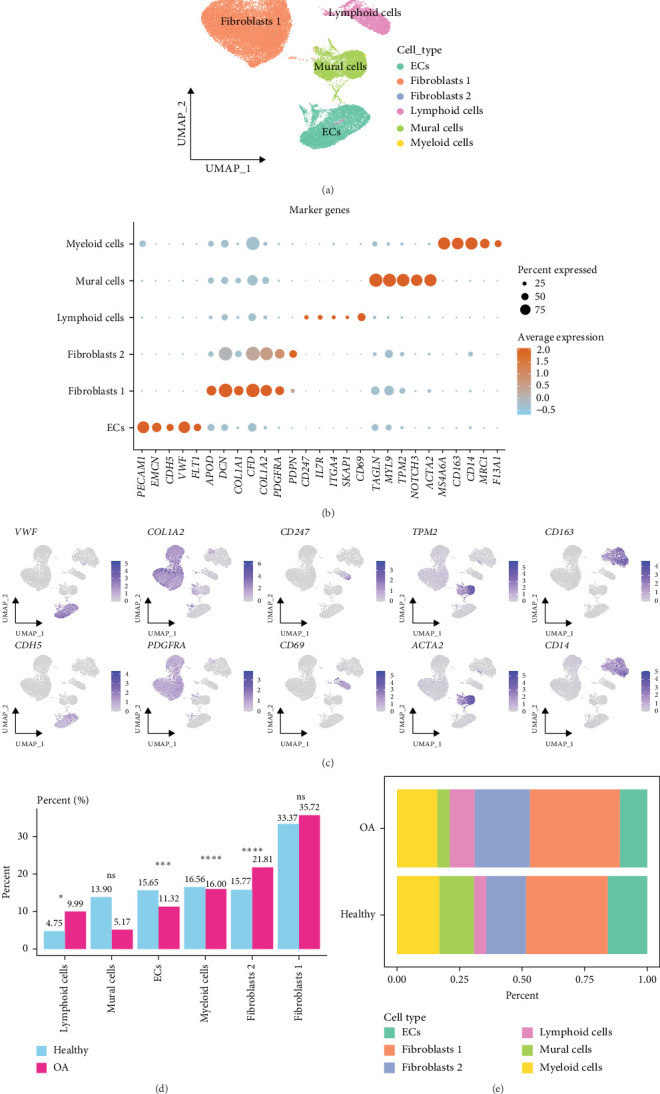
Single-cell atlas of osteoarthritis (OA). (A) The distribution of different cell types visualized by UMAP plot. (B) The expression levels of marker genes in different cell types displayed by bubble plot. (C) The expression patterns of key genes on UMAP map. (D) The percent of different cell types in OA and healthy samples. (E) Relative proportion of different cell types in OA and healthy samples. *⁣*^*∗*^*p* < 0.05, *⁣*^*∗∗∗*^*p* < 0.001, *⁣*^*∗∗∗∗*^*p* < 0.0001; ns, no significant difference.

Cell filtering, standardization, and dimensionality reduction clustering were performed on the scRNA-seq data of GSE216651. A total of 63,784 cells were acquired, and six cell clusters were identified, namely, endothelial cells (ECs), two different subtypes of fibroblasts (Fibroblast 1 and Fibroblast 2), lymphoid cells, mural cells, and myeloid cells (Figure 1A). Based on marker gene annotation, cell clusters were identified as follows: myeloid cells high-expressed *CD163* and *CD14*, etc.; mural cells high-expressed *TAGLN*, *MYL9*, *TPM2*, etc.; lymphoid cells high-expressed *CD69*; Fibroblast 2 high-expressed *PDPN*; Fibroblast 1 high-expressed *APOD*, *DCN*, and *COL1A1*, etc.; ECs high-expressed *PECAM1* and *EMCN*, etc. (Figure 1B,C). Moreover, comparison of the proportions of different cell clusters between the OA sample and healthy samples demonstrated that the number of fibroblasts (35.72% Fibroblast 1, 21.81% Fibroblast 2) and lymphoid cells (9.99%) in the OA patients was higher than those in the healthy patients (33.37% Fibroblast 1, 15.77% Fibroblast 2, 4.75% lymphoid cells), with fibroblasts accounting for the highest proportion among all the cell clusters (Figure 1D,E).

### 3.2. Heterogeneity of Fibroblasts in OA

**Figure 2:**
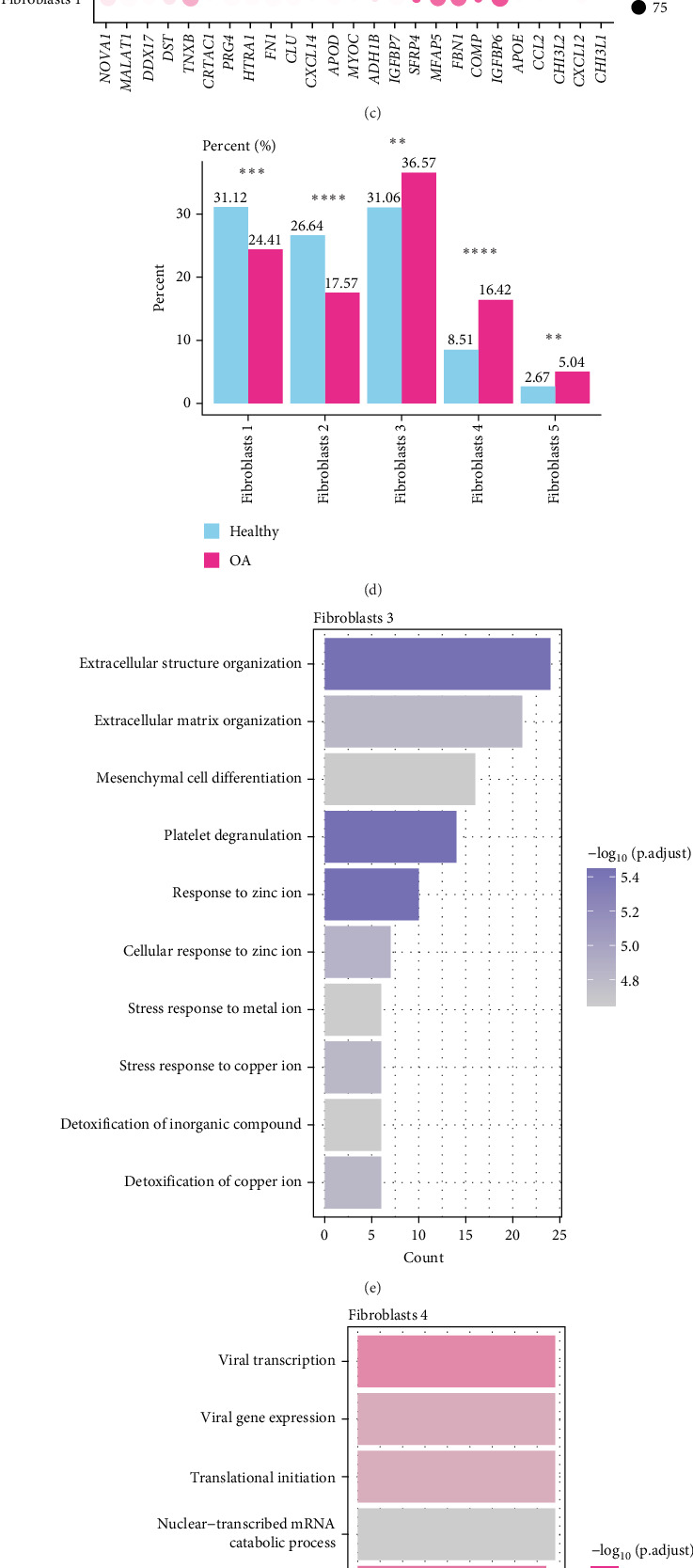
The heterogeneity of fibroblasts. (A) The differences in biological processes (BPs) enriched in OA and healthy samples. (B) The classification of fibroblasts subtypes displayed by UMAP plot. (C) The high-expressed marker genes in different fibroblasts subtypes. (D) The percent of different fibroblasts subtypes in OA and healthy samples. (E) The BPs enriched in Fibroblasts 3. (F) The BPs enriched in Fibroblasts 4. (G) The BPs enriched in Fibroblasts 5. *⁣*^*∗∗*^*p* < 0.01, *⁣*^*∗∗∗*^*p* < 0.001, *⁣*^*∗∗∗∗*^*p* < 0.0001.

To further evaluate the cell types that contributed to OA fibrosis, we focused on exploring the potential impact of fibroblasts on OA. GSEA revealed significant enrichment of fibrosis-related GO-BP terms in OA samples, including collagen fibril organization, extracellular matrix organization, and supramolecular fiber organization (Figure 2A). As shown in the UMAP plot, fibroblasts were reclustered to five cell subpopulations (Fibroblast 1–5) (Figure 2B), which were marked as Fibroblast 1 (*SFRP4*, *MFAP5*, *FBN1*, *COMP*, *IGFBP6*), Fibroblast 2 (*CXCL14*, *APOD*, *MYOC*, *ADH1B*, *IGFBP7*), Fibroblast 3 (*CRTAC1*, *PRG4*, *HTRA1*, *FN1*, *CLU*), Fibroblast 4 (*APOE*, *CCL2*, *CHI3L2*, *CXCL12*, *CHI3L1*), and Fibroblast 5 (*NOVA1*, *MALAT1*, *DDX17*, *DST*, *TNXB*) (Figure 2C). Notably, the proportion of Fibroblast 3 (36.57%), Fibroblast 4 (16.42%), and Fibroblast 5 (5.04%) in the OA samples was markedly higher than the healthy samples (31.06% Fibroblast 3, 8.51% Fibroblast 4, 2.67% Fibroblast 5), with Fibroblast 3 accounting for the highest proportion (Figure 2D). Furthermore, enrichment analysis showed that the fibrosis-related pathways, such as extracellular matrix organization, mesenchymal cell differentiation, and response to metal ions, were noticeably enriched in Fibroblast 3 (Figure 2E), and that Fibroblast 3 may be a main contributor to OA fibrosis. The pathways associated with cellular protein synthesis and turnover, such as protein localization to endoplasmic reticulum (ER), SRP-dependent cotranslational protein targeting to membrane, and protein targeting to ER, were mainly enriched in Fibroblast 4 (Figure 2F), indicating that it may be in a state of high metabolic activity or stress response. Moreover, pathways related to RNA splicing were notably associated with Fibroblast 5 (Figure 2G), indicating that Fibroblast 5 was closely involved in mRNA post-transcriptional modification.

### 3.3. The Role of Fibroblast 3 in OA Development Was Analyzed Using Pseudo-Time Trajectory Analysis

**Figure 3:**
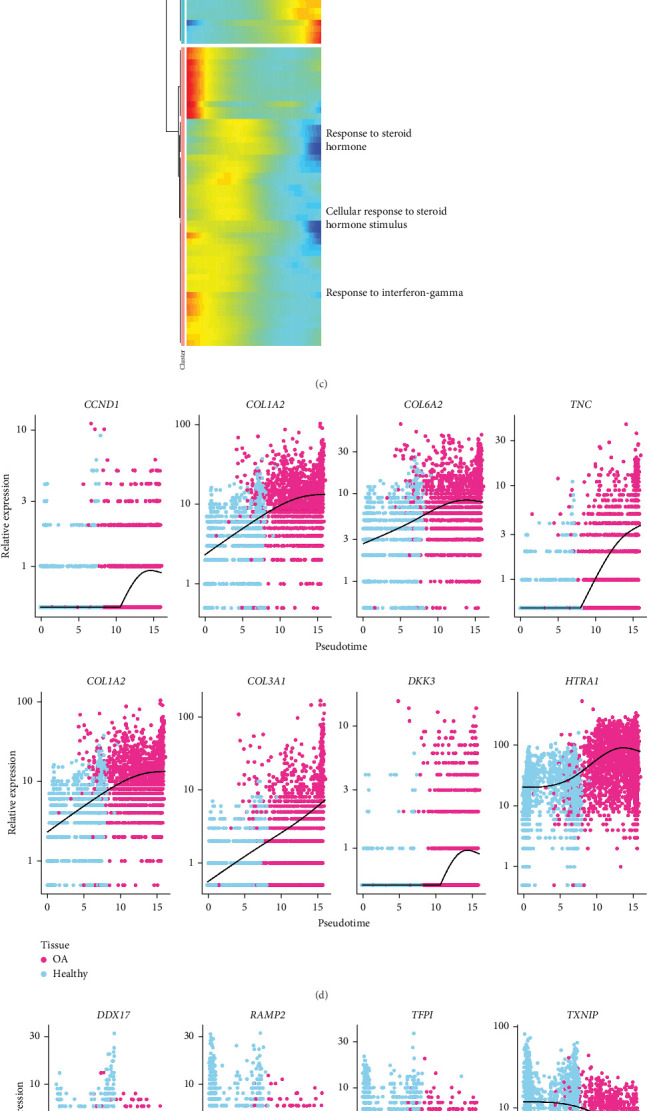
The pseudotime trajectory analysis of Fibroblasts 3. (A,B) The differentiation trajectory of Fibroblasts 3 from healthy tissue to OA. (C) Heatmap of the expression levels of genes related pseudotime and involved pathways. (D) Scatterplot of gene expression levels related to extracellular matrix and cell adhesion. (E) Scatterplot of gene expression levels associated with response to steroid hormone and response to interferon-gamma.

The role of Fibroblast 3 in the progression of OA was further explored by pseudotime trajectory analysis, with the healthy tissue on the right side as the starting point and OA tissue on the left side as the endpoint (Figure 3A,B). Analysis on the dynamic changes in genes during OA development demonstrated that the upregulated genes were primarily enriched in PI3K-Akt signaling pathway, ECM–receptor interaction, collagen metabolic process, and response to transforming growth factor β, while the downregulated genes were mainly enriched in response to steroid hormone, cellular response to steroid hormone stimulus, and response to interferon-gamma (Figure 3C). The pseudotime trajectory showed that *CCND1*, *COL1A2*, *COL6A2*, *TNC*, *COL3A1*, *DKK3*, *HTRA1* were related to extracellular matrix and cell adhesion and exhibited a significant upregulation expression trend during the transformation of healthy cells to OA (Figure 3D), while *DDX17*, *RAMP2*, *TFPI*, *TXNIP*, *DAPK1*, *GSN*, *HLA-A*, *HLA-C* were associated with response to steroid hormone and response to interferon-gamma and showed a downregulated expression trend (Figure 3E).

### 3.4. Four Diagnostic Biomarkers of OA Were Identified and Exhibited Strong Diagnostic Performance

**Figure 4:**
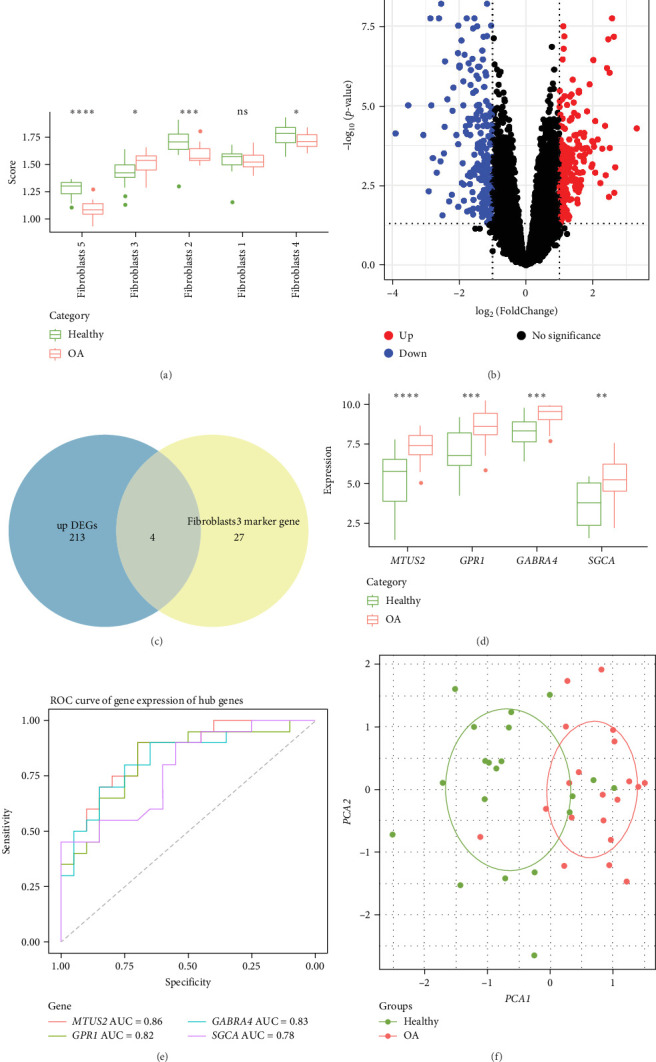
Identification of diagnostic biomarkers for OA. (A) The enrichment score of each fibroblasts subtype of OA and healthy samples in the independent datasets. (B) Volcano plot of differentially expressed genes (DEGs) between OA and healthy samples in the independent datasets. (C) Venn diagram of upregulated DEGs and Fibroblasts 3 marker genes. (D) The expression levels of biomarker genes in OA and healthy samples in the independent datasets. (E) ROC curves of biomarker genes in the independent datasets. (F) PCA plot of key genes expression levels based on the independent datasets. *⁣*^*∗∗∗∗*^*p* < 0.0001, *⁣*^*∗∗∗*^*p* < 0.001, *⁣*^*∗∗*^*p* < 0.01, *⁣*^*∗*^*p* < 0.05; ns, not significant.

The role of Fibroblast 3 in the progression of OA was validated in the combined validation dataset of the GSE55457 and GSE55235 datasets. The ssGSEA results showed that compared to the healthy group, the enrichment scores of Fibroblast 2, Fibroblast 4, and Fibroblast 5 were significantly lower in OA group, while that of Fibroblast 3 was notably higher in the OA group (Figure 4A), indicating that Fibroblast 3 may promote the development of OA and can serve as a predictive factor for OA. Subsequently, 197 downregulated and 217 upregulated DEGs were collected between healthy and OA samples in the independent datasets (Figure 4B). Then, four genes in the intersection between the 217 upregulated DEGs and the 31 marker genes in Fibroblast 3 were obtained as the diagnostic biomarkers for OA (Figure 4C). Furthermore, the diagnostic performance of the four biomarkers in OA was validated in the independent datasets, and we found that the expression of *MTUS2*, *GPR1*, *GABRA4*, and *SGCA* was significantly upregulated in OA samples (Figure 4D). The ROC curve showed that the AUC of *MTUS2*, *GPR1*, *GABRA4* and *SGCA* reached 0.86, 0.82, 0.83, and 0.78, respectively (Figure 4E), which proved that these biomarkers could effectively predict OA. According to PCA, cell clusters can distinguish between OA tissues and normal tissues (Figure 4F).

## 4. Discussion

OA is a progressive and degenerative joint disease primarily caused by factors such as age, gender, obesity, genetics, and joint injury [25]. This study identified five fibroblast subtypes (Fibroblast 1–5) by systematic analysis based on the scRNA-seq data in OA and found that the proportion of Fibroblast 3 was significantly higher in OA tissues and it was enriched with fibrosis-related pathways such as extracellular matrix remodeling and mesenchymal cell differentiation. The pseudotime trajectory analysis further revealed the dynamic expression changes of Fibroblast 3 involved in fibrosis during OA progression. Combining two independent bulk transcriptome datasets, we screened four diagnostic biomarkers (*MTUS2*, *GPR1*, *GABRA4*, and *SGCA*), which can effectively distinguish OA from normal tissues. The above results revealed an important role of fibroblasts in the pathogenesis of OA and also provided new perspectives for exploring the pathogenic functions of their subtypes. As the first study to integrate single-cell subpopulation identification with the validation of external data, this work systematically explored the key cell subtypes of OA and their molecular characteristics. Our present discoveries provided a theoretical basis and potential targets for the early diagnosis and treatment of OA patients and also showed a potential for clinical application.

Based on previous studies, *MTUS2* plays a critical role in cytoskeletal regulation and microtubule assembly and dynamics and is involved in the development of various types of cancers and Alzheimer's disease (AD) [26, 27]. *MTUS2* protein localizes to microtubule plus-ends and contributes to cytoskeletal remodeling and cell migration, potentially influencing the tissue microenvironments. Our study was the first to report significantly upregulated *MTUS2* in OA tissues and its potential involvement in extracellular matrix remodeling and fibroblast-mediated fibrosis during OA progression.

*GPR1*, a G protein-coupled receptor, is primarily expressed in adipose tissue and skeletal muscle and functions as a receptor for adipokine chemerin. It is implicated in various physiological and pathological processes, including adipocyte differentiation, energy metabolism, inflammation, and angiogenesis [28–30]. Our findings revealed aberrant expression of *GPR1* in OA samples, showing its potential role in regulating local inflammation and vascular remodeling within the microenvironment of OA.

*GABRA4* encodes the α4 subunit of the GABAA receptor, a key component of the inhibitory neurotransmitter GABA system. Mutations in *GABRA4* are associated with autism spectrum disorders (ASDs) [31]. Knockout mouse models exhibit altered neural plasticity and resistance to seizures [32]. While most existing studies focused on its role in the nervous system, we observed the upregulation of *GABRA4* in OA synovial tissues, suggesting a potential immunoregulatory or neuro–synovial interaction role in joint pathology, which, however, still requires further investigation.

*SGCA*, a key component of the sarcoglycan complex within the dystrophin-associated protein complex (DAPC), encodes α-sarcoglycan and plays a crucial part in maintaining muscle fiber integrity. Mutations in *SGCA* are known to be able to cause limb-girdle muscular dystrophy type 2D (LGMD2D) [31], and *SGCA* has also been included in a 16-gene signature developed by computational analysis for distinguishing rheumatoid arthritis from OA [33]. In our study, *SGCA* is identified as a high-expressed marker gene of Fibroblast 3, suggesting the involvement of *SGCA* in OA pathogenesis by mediating synovial responses to mechanical stress. Taken together, although previous studies have not directly linked *MTUS2*, *GPR1*, or *GABRA4* with OA, our integrated analysis combining single-cell and bulk transcriptomic data was the first to identify and validate these four genes as potential diagnostic biomarkers based on fibroblast subtypes. These findings offered novel insights into OA pathogenesis and provided a foundation for future studies to explore their mechanistic roles and therapeutic value.

However, there were some limitations in our study. First, a relatively small sample size of the scRNA-seq data in this study limited a comprehensive characterization of fibroblast heterogeneity in OA patients. Therefore, future study could expand the number of samples and include samples of different ages, genders, OA stages, and joint sites to enhance the statistical reliability and clinical applicability of our results. Furthermore, although scRNA-seq analysis classified cell subtypes, it failed to show the spatial distribution of fibroblasts in synovial tissues, which limited the understanding of cell-microenvironment interactions. In the future, we will combine spatial transcriptome technology to examine the localization of Fibroblast 3 in the synovial microenvironment and its interactions with immune cells and vascular structures. Finally, the key fibroblast subtypes and 4 diagnostic genes identified in this study have not been validated by in vitro or in vivo experiments, which limited their clinical application value. Therefore, the expression of these key genes needs to be verified at the tissue and cellular levels employing immunohistochemical staining, qRT-PCR, and western blot, and their specific roles in the function of fibroblasts should be explored using functional experiments.

## 5. Conclusion

In summary, this study identified five fibroblast subtypes for OA based on scRNA-seq data, among which Fibroblast 3 was significantly enriched in OA tissues and associated with fibrosis-related pathways. Pseudotime trajectory analysis revealed persistent activation of Fibroblast 3 during OA progression, indicting its key role in synovial fibrosis. In addition, we screened four diagnostic markers (*MTUS2*, *GPR1*, *GABRA4*, and *SGCA*) strong discriminatory capability for OA. These findings collectively revealed an important role of fibroblasts in the pathogenesis of OA and provided potential diagnostic targets for the intervention of OA.

## Data Availability

The datasets generated and/or analyzed during the current study are available in the (GSE216651) repository, (https://www.ncbi.nlm.nih.gov/geo/query/acc.cgi?acc=GSE216651), (GSE55457) repository, (https://www.ncbi.nlm.nih.gov/geo/query/acc.cgi?acc=GSE55457) and (GSE55235) repository, (https://www.ncbi.nlm.nih.gov/geo/query/acc.cgi?acc=GSE55235).

## Nomenclature

AUC: Area under the ROC curves
BP: Biological processes
DEGs: Differentially expressed genes
ECs: Endothelial cells
ER: Endoplasmic reticulum
FC: FoldChange
GEO: Gene Expression Omnibus
GO: Gene Ontology
GSEA: Gene set enrichment analysis
OA: Osteoarthritis
PCA: Principal component analysis
ROC: Receiver operating characteristic
scRNA-seq: Single-cell RNA-sequencing
ssGSEA: Single sample GSEA
UMAP: Uniform manifold approximation and projection.
